# Gestational Diabetes Mellitus (GDM) Risk for Declared Family History of Diabetes, in Combination with BMI Categories

**DOI:** 10.3390/ijerph18136936

**Published:** 2021-06-28

**Authors:** Małgorzata Lewandowska

**Affiliations:** 1 Medical Faculty, Lazarski University, 02-662 Warsaw, Poland; mal2015lewandowska@gmail.com; 2 Division of Gynecological Surgery, University Hospital, 60-535 Poznan, Poland

**Keywords:** gestational diabetes mellitus, family history, paternal diabetes, maternal diabetes, grandparents, obesity, overweight, underweight

## Abstract

Whether categories of family history of diabetes can act as independent risk factors for gestational diabetes mellitus (GDM-1, -2) has not yet been established, and neither has it been established how categories of body mass index (BMI) affect these relationships. A group of 912 women without chronic diseases, recruited in the first trimester, was investigated: 125 (13.7%) women developed GDM-1 (treated with diet); 21 (2.3%) women developed GDM-2 (treated with insulin); and a control group consisted of 766 non-diabetic women. A multiple logistic regression was used to evaluate adjusted odds ratios (AOR (95% confidence intervals)) of GDM-1 and GDM-2 for declared diabetes in the parents or grandparents (separately). These relationships were investigated in the whole cohort and subgroups of pre-pregnancy BMI. (1) Some categories of the family history were independent risk factors of GDM-1 or GDM-2. Compared to ‘absence of diabetes in the family’, women with diabetes in the father had a 3.68-fold increase in GDM-1 risk (AOR-b = 3.68 (2.23–6.07)), and women with diabetes in the mother had a 2.13-fold increase in GDM-1 risk (AOR-b = 2.13 (1.1–4.14)) and a 4.73-fold increase in GDM-2 risk (AOR-b = 4.73 (1.26–17.77)). Women with diabetes in the grandmother had a 2.34-fold increase in GDM-1 risk (AOR-b = 2.34 (1.29–4.24)). (2) The cumulative assessment of diabetes in the parents and/or grandparents was not related to the intensification of the risk of GDM, except for the increased risk of GDM-1 for diabetes in both mother and grandmothers simultaneously (AOR-b = 8.80 (1.16–66.57)), however, this group was very small. (3) The analyses in the subgroups of BMI categories showed that diabetes in the father was also an independent risk factor of GDM in the subgroup of pregnant women with normal BMI. In the subgroups of overweight and/or obesity, the risk of GDM for paternal and maternal diabetes was approximately twice as high as compared to the results of pregnant women with normal BMIs. Additionally, apart from the maternal influence of diabetes, the results suggest a significant influence of diabetes in the father on the risk of GDM, even (interestingly) in lean pregnant women.

## 1. Introduction

Gestational diabetes mellitus (GDM) is a pregnancy-specific disease defined as ‘diabetes that is first diagnosed in the second or third trimester of pregnancy that is clearly not overt diabetes’ (using newer definition in accordance with the guidelines of the American Diabetes Associations, 2021) [1,2,3,4,5]. This disease includes GDM-1 (treated with diet) and GDM-2 (when required, treated by adding insulin to the diet in order to normalize glycemia) [2]. GDM is an important public health issue. Globally, on average, 16% of pregnancies develop GDM (i.e., average 18 million women) depending on the region and diagnostic criteria [4,6,7], and 80–90% of cases develop GDM-1 (when diet treatment was sufficient to normalize glycemia) [3]. Importantly, this disease is associated with both short-term (perinatal) and long-term complications in the mother and the newborn. Long-term complications include an increased risk of developing type 2 diabetes (T2D) and obesity later in the mother’s and baby’s life, revealing the adverse intergenerational effects of GDM [4,8,9,10]. This is related to the theory of Developmental Origins of Health and Disease (DOHaD) [11,12]. Early identification of GDM risk allows for early interventions to reduce maternal and child complications [4,13].

Certain maternal characteristics have been linked to the development of gestational diabetes mellitus (GDM), such as obesity, GDM in earlier pregnancies, older maternal age, ethnicity and multiparity as well as family history of diabetes [9,14,15,16,17,18,19,20,21,22,23,24,25]. The relationship between a family history of diabetes and GDM reflects the influence of genetic and environmental conditions and highlights the multifactorial nature of the pathophysiology of GDM. There are publications describing the genetic and epigenetic etiology of GDM and the association of GDM with some type 2 diabetes risk (T2D) gene polymorphisms [26,27,28,29,30,31].

However, it has not been established whether categories of the family history of diabetes can act as independent risk factors for gestational diabetes mellitus (GDM). No studies were found for risk of GDM-1 and -2 separately. In previous studies, discrepancies in results were found. These studies showed both similar and different effects of paternal and maternal diabetes on the risk of GDM [32,33,34]. Systematic reviews (by Lee et al. and by Moosazadeh et al.) showed discrepancies in the results of GDM risk for ‘family history of diabetes’ as a single variable [24,35]. In the literature, the discrepancies in sample sizes, ethnicity, the definitions of ‘family diabetes’ or the degree of adjustment were found, and the diagnostic criteria for GDM were different. It has not been established how categories of maternal body mass index (BMI) affect the relationships in question. Obesity is a known risk factor of GDM [18,19,36], and currently, there is an epidemic of obesity [4,37].

The main objective of this study was to evaluate eleven (11) categories of declared family history of diabetes (evaluating separately diabetes in the father, mother, grandmother and grandfather as well as combinations of these categories) as risk factors for gestational diabetes mellitus (GDM-1 and GDM-2 separately) in a prospectively pooled cohort of pregnant women. An additional aim was to investigate these relationships in the subgroups of categories of pre-pregnancy BMI. We have not found such a study in the available literature.

## 2. Materials and Methods

This study was based on the data from a prospective cohort of pregnant women recruited in 2015 and 2016. The Research Center was located at the Obstetrics and Gynecology Hospital of the Poznan University of Medical Sciences (Poland), where the number of births reaches 6000–8000 per year.

### 2.1. Ethics

This study was conducted in accordance with the Helsinki Declaration, and the Bioethics Committee of the Poznan University of Medical Sciences approved this research project (Number 769/15). The participation this study was voluntary. All women signed an informed consent form prior to the study.

### 2.2. Inclusion Criteria

The cohort under investigation consisted of pregnant women who were recruited at the end of the first trimester (according to the relevant criteria). In the second stage of the study (after childbirth), the pregnancy results were taken from the medical records and analyzed.

The inclusion criteria were as follows: pregnant women of Caucasian race from the Wielkopolska region, aged 18–45 years (calculated at the conception date) and singleton pregnancy at 10–14 weeks (for the recruitment date), childbirth without defects in the ≥25th week, as well as the lack of chronic diseases, except for abnormal body index mass (BMI) (including the lack of pre-existing diabetes, hypertension, immuno-logical and/or inflammatory diseases or kidney and liver diseases). These criteria included primiparous and multiparous women who, among other things, either had or did not have a family history of diabetes (and other diseases) and who either had or did not have gestational diabetes in previous pregnancies.

### 2.3. Methods

The place of recruitment (in the 10th–14th weeks) was the Central Laboratory in the Research Center. Information about the study was posted in a prominent place and was directed to all women who came to the laboratory for the standard tests. The women who declared their willingness to participate in the study completed a questionnaire (independently, in the presence of experienced midwives). The questionnaire included questions about sociodemographic data, the participant’s obstetric history and family history of diseases. The participants declared their own weight before pregnancy and gave information on stimulants (smoking, drinking alcohol and others) and medications used before pregnancy and in the first trimester, including the use of vitamin and micronutrient preparations typical for pregnancy. The participants denied having consumed alcohol during pregnancy.

In the second stage of the study (postpartum), data on the outcomes of pregnancy, including maternal complications and unfavorable results of the newborn, were taken from the medical records. At the same time, some data included in the medical records of the pregnant woman were verified with the data in the Questionnaire (e.g., information on the family history of chronic diseases or smoking). Additionally (after the 12th week of puerperium), an additional questionnaire was completed regarding the health of the mother in puerperium (participants were contacted by phone or e-mail).

A total of 1300 women meeting the recruitment criteria in week 10–14 declared their willingness to participate in this project, and all of them completed the questionnaire. After the end of the second stage of the study, 388 women were excluded due to incomplete data (*n* = 340) as well as due to childbirth with a defect, delivery before the 25th week of pregnancy, diagnosis of diabetes before the 18th week of pregnancy or diagnosis of hypertension before the 20th week, development of thrombotic disorders and infections requiring hospitalization or due to the lack of cooperation (*n* = 48).

Ultimately, the original cohort was 912 women; 766 women were non-diabetic (controls) and 146 (16%) women developed gestational diabetes mellitus (GDM), including 125 (13.7%) women with GDM-1 and 21 (2.3%) women with GDM-2.

### 2.4. Sample Size

The main goal of this analysis was to evaluate the associations between family diabetes history criteria and the risk of GDM-1 and GDM-2.

The minimum sample size was calculated using the formula for a single proportion (for 95% confidence intervals and *α* = 5%; *Z* = 1.962: critical value of normal distribution at *α*/2; ‘*p*’: sample proportion; *d*’: margin of error):

(1) $$n=Z_{\frac{\alpha }{2}\;}^{2}\frac{p\cdot \left(1-p\right)}{d^{2}}$$

For the error value of d = 0.03 (3%), the minimum sample size was: 574 for the proportion *p* = 0.16 (16%) (for all cases of GDM in this study); 506 for the proportion *p* = 0.137 (13.7%) (for cases of GDM-1); 96 for the proportion *p* = 0.023 (2.3%) (for cases of GDM-2). The minimum sample size was 802 for the proportion *p* = 0.25 (25%) (the highest mean proportion found in the literature) [4,6,7].

This cohort (*n* = 912) was good enough to discover the relationships studied.

### 2.5. Definitions of Dependent Variables

In this analysis, cases of gestational diabetes mellitus (GDM) were investigated as dependent variables. The diagnosis of GDM was consistent with the following definition: ‘diabetes that is first diagnosed in the second or third trimester of pregnancy that is clearly not overt diabetes’ (in accordance with the guidelines of the American Diabetes Associations, Standards of Medical Care, 2021) [1].

The diagnosis of GDM was based on the two-hour 75 g oral glucose tolerance test (75 g OGTT) at the 24th–28th gestational weeks, as recommended by the World Health Organization (WHO) 2013. Criteria used in all study centres were consistent throughout the timescale of this study.

Undiagnosed, pre-existing diabetes were excluded based on the following procedures. In women without risk factors, fasting glucose levels are measured at the beginning of pregnancy/during the first visit to the gynecologist (the normal result is < 92 mg/dL). In women with risk factors, the 75 g OGTT test is performed at the beginning of pregnancy/during the first visit to the gynecologist. Normal results allow you to plan the test (75 g OGTT) in week 24–28 or when symptoms suggestive of diabetes appear.

GDM was diagnosed if one or more of the following criteria are met: fasting plasma glucose 5.1–6.9 mmol/L (92–125 mg/dL); a 1-h plasma glucose following a 75 g oral glucose load ≥10.0 mmol/L (180 mg/dL); or a 2-h plasma glucose following a 75 g oral glucose load 8.5–11.0 mmol/L (153–199 mg/dL).

GDM-1 was diagnosed when diet treatment was sufficient to normalize glycemia, and GDM-2 was diagnosed when treatment required adding insulin to the diet in order to normalize glycemia [2,3,5]. If plasma glucose levels were over the therapeutic range (fasting ≥91 mg/dL and 1 h after meal ≥140 mg/dL), treatment with insulin was initiated. No other drugs (e.g., metformin) were used.

According to the supplementary questionnaire data (collected after the 12th week of puerperium), there were no new cases of diabetes diagnosed up to 12 weeks postpartum. In this analysis, detailed information on GDM (e.g., the degree of glycemic control during pregnancy) was not known for each case (data were extracted from medical records of women admitted to childbirth).

The gestational age was determined using ultrasound and crown-rump length was assessed between the 10th and 13th (+6 days) week.

### 2.6. Independent Variables

Categories of the family history of diabetes were assessed as independent variables. The family’s history was part of the maternal characteristics. The family history was self-reported in the Questionnaire and women answered the question:

‘Who in the family suffers from these chronic diseases: diabetes, hypertension, myocardial infarction, coronary artery disease, stroke, tumors or other diseases (describe the details)’.

Participants of the study entered family members for the diseases mentioned (as well as described details, e.g., the forms of the diseases). In most cases, details of the history were not known (e.g., no information on the type of diabetes, T1D or T2D).

The information (from the questionnaires) was also verified with the data from the medical records. The information on the family history of diabetes in the father (*n* = 110), mother (*n* = 70), grandmother (s) (*n* = 93), grandfather (s) (*n* = 43), sister (s) (*n* = 4) and brother (s) (*n* = 2) were found. The information was inconclusive in two cases (missing data). Information on the presence of GDM in previous pregnancies (applies to pregnant women) (*n* = 11) as well as the presence of GDM in pregnancies of the mothers and sisters (*n* = 8) was also collected.

This study investigated the following eleven (11) categories of diabetes in family: (1) diabetes in the mother only, (2) diabetes in the father only, (3) diabetes in the father or mother, (4) diabetes in both parents simultaneously, (5) diabetes in the grandmother(s) only, (6) diabetes in the grandfather(s) only, (7) diabetes in the grandfathers or grandmothers, (8) diabetes in both grandparents simultaneously, (9) diabetes in both mother and grandmothers simultaneously, (10) diabetes in both father and grandfathers simultaneously, (11) absence of diabetes in the family (in the parents and other relatives). The reference category was ‘absence of diabetes in the family’.

### 2.7. Covariates

In this analysis, the following covariates (risk factors for gestational diabetes mellitus, GDM) were included: multiparity, pre-pregnancy BMI, maternal age, gestational weight gain (GWG) out of the range of the Institute of Medicine (IOM) recommendation 2009 and smoking in the first trimester (Model-a), plus prior GDM (Model-b) [9,14,15,16]. Among smoking categories, ‘smoking in the first trimester’ was a confounder, according to results from previous studies [20].

### 2.8. Statistical Analyses

Statistical analyses were conducted using Statistica software (Version 13) (TIBCO, Palo Alto, CA, USA). Maternal characteristics (basic risk factors and family history of diabetes) were compared between women who developed gestational diabetes mellitus (GDM) (case group) and non-diabetic women (control group) and were described by the percentage (%) or using median and 25–75% interquartile ranges (IQR).

The Shapiro–Wilk test was used to investigate the normality of the data distribution. Because continuous variables were not normally distributed, the Mann–Whitney U test was applied for comparisons of these variables. The Pearson chi-square test (or Fisher exact test when Cochran assumption was not met) was applied for comparisons of binomial variables. The Cochran–Armitage test was applied for comparisons of trend for categorical variables. The *p*-value < 0.05 was assumed to be significant.

Single (OR) and multiple (AOR) logistic regressions were used to calculate the odds ratios (with 95% confidence intervals) of gestational diabetes GDM-1 and GDM-2 for each category of family history of diabetes in relation to the reference category ‘the lack of diabetes in the family’ (OR = 1.00). The results obtained in multiple logistic regression were adjusted for the adopted covariates (Table 1). The Wald test was applied to calculate *p*-value (*p* < 0.05 was assumed to be significant).

These relationships were investigated in the whole cohort and subgroups of pre-pregnancy BMI categories (as well as smoking categories).

## 3. Results

### 3.1. Basic Characteristics of the Partcipants

Table 2 shows the basic characteristics of participants developing gestational diabetes mellitus (GDM) (cases). Women developing GDM (vs. non-diabetic women) had a statistically significantly higher median age and pre-pregnancy body mass index (BMI). There were significantly more women in the GDM group with pre-pregnancy obesity (21.9% vs. 8.6%). There were significantly more women in the GDM group (vs. non-diabetic women) with prior GDM (5.5% vs. 0.4%).

Newborn outcomes also differed: in the GDM group (vs. non-diabetic women), a statistically significantly lower gestational age was found, and the percentage of macrosomia cases was higher (14.4% vs. 9.9%).

Table 3 shows the risk of GDM-1 and GDM-2 for basic risk factors (calculated in multiple logistic regression). The set of data (with the number of cases/controls and raw odds ratios) is presented in the supplemental Table S1.

The highest GDM-1 and GDM-2 risks were found for prior GDM. Multiparity (vs. primiparity) was associated with a higher risk of GDM-1. The oldest age (≥40 years) was associated with higher odds ratios of GDM-1 and -2. Gestational weight gain (GWG) below the range was associated with a higher risk of GDM-1 and -2, however, reduced GWG is more likely to be a consequence of GDM diagnosis, rather than a contributing cause.

Among modifiable factors, pre-pregnancy obesity was associated with a higher risk of GDM-1 and GDM-2 (comparing to normal BMI). Smoking in the first trimester was associated with a higher odds ratio of GDM-1, however, this subgroup was small (Table 2).

Table 4 shows the basic characteristics of declared family history of diabetes in the case and control groups. In the GDM group (compared to non-diabetic group), a statistically significantly higher frequency of women with declared diabetes in the father (*p* < 0.001), mother (*p* = 0.019) and grandmother(s) (*p* = 0.030) was found.

### 3.2. Risk of GDM (-1 and -2) for Diabetes in the Parents and Grandparents

Table 5 shows the adjusted risk of gestational diabetes mellitus (GDM, -1 and -2) for a declared family history of diabetes in the parents (calculated in multiple logistic regression).

The results show that diabetes in the father or mother were the independent risk factors of GDM-1, and diabetes in the mother was the risk factor of GDM-2 (the results were sustained after adjusted for multiparity, pre-pregnancy BMI, maternal age, gestational weight gain (GWG) out of the range and smoking in the first trimester and prior GDM).

Compared to ‘absence of diabetes in the family’, women with diabetes in the father had a 3.68-fold increase in GDM-1 risk (AOR-b = 3.68 (2.23–6.07)), and women with diabetes in the mother had a 2.13-fold increase in GDM-1 risk (AOR-b = 2.13 (1.1–4.14)). Compared to ‘absence of diabetes in the family’, women with diabetes in the mother had a 4.73-fold increase in GDM-2 risk (AOR-b = 4.73 (1.26–17.77)).

In sum, apart from the maternal influence of diabetes, the results in Table 5 suggest a significant influence of diabetes in the father on the risk of GDM (this is seen especially in the risk analysis for all GDM cases).

The cumulative assessment of diabetes in the father and/or mother was not related to the intensification of the risk of GDM.

Table 6 shows the risk of GDM (-1 and -2) for the family history of diabetes in the grandparents. Compared to no family history of diabetes, only women with diabetes in the grandmother had statistically significant increase in GDM-1 risk (AOR-b = 2.34 (1.29–4.24)). The cumulative assessment of diabetes in the grandparents was not related to the intensification of the risk of GDM. The risk score profile for all cases of GDM was similar to the score profile for GDM-1.

Table 7 shows the risk of GDM (-1 and -2) for cumulative assessment of diabetes in the parents and grandparents. The analysis showed that the risk of GDM-1 was slightly higher for declared diabetes in the father or grandfathers (AOR-b = 3.05 (1.9–4.88)) than diabetes in the mother or grandmothers (AOR-b = 2.14 (1.31–3.50)). However, these results were not higher than the risk assessed for diabetes in the father or (separately) mother.

Interestingly, women with diabetes in both mother and grandmothers simultaneously had an 8.80-fold increase in GDM-1 risk (AOR-b = 8.80 (1.16–66.57)), however, this group (the number of cases and controls) was very small, and this result had a wider confidence interval (CI).

The risk score profile for all cases of GDM was similar to the score profile for GDM-1.

**Figure 1:**
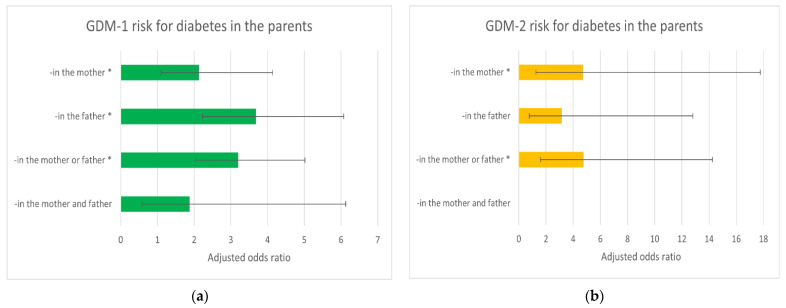
The adjusted odds ratios (model-b) of gestational diabetes mellitus GDM-1 (**a**) and GDM-2 (**b**) for declared diabetes in the parents (compared to ‘absence of diabetes in the family’). These results were calculated in multiple logistic regression after adjusted for multiparity, pre-pregnancy BMI, maternal age, gestational weight gain (GWG) out of the range, smoking in the first trimester and prior diabetes in pregnancy. * Statistically significant (Table 5). GDM-1: gestational diabetes mellitus treated with diet (*n* = 125); GDM-2: gestational diabetes mellitus treated with insulin (*n* = 21); Controls: non-diabetic women (*n* = 766). Horizontal lines represent (95%) confidence intervals (CI) (Table 5).

**Figure 2:**
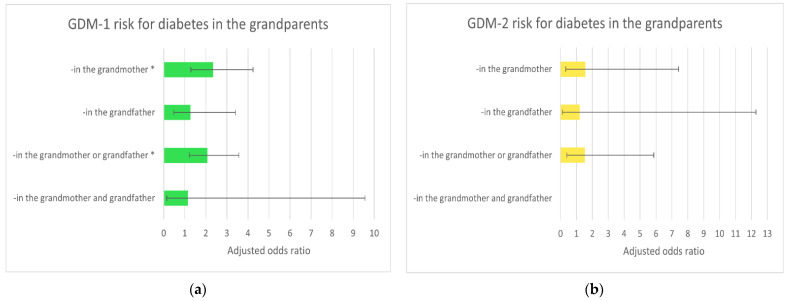
The adjusted odds ratios (model-b) of gestational diabetes mellitus GDM-1 (**a**) and GDM-2 (**b**) for declared diabetes in the grandparents (compared to ‘absence of diabetes in the family’). These results were calculated in multiple logistic regression after adjusted for multiparity, pre-pregnancy BMI, maternal age, gestational weight gain (GWG) out of the range, smoking in the first trimester and prior diabetes in pregnancy. * Statistically significant (Table 6). GDM-1: gestational diabetes mellitus treated with diet (*n* = 125); GDM-2: gestational diabetes mellitus treated with insulin (*n* = 21); Controls: non-diabetic women (*n* = 766). Horizontal lines represent (95%) confidence intervals (CI) (Table 6).

Figure 1 and Figure 2 show the relationship between diabetes (GDM-1 and -2) risk and declared family history of diabetes in the parents (Figure 1) and grandparents (Figure 2). Diabetes in the father, mother and grandmothers were independent (*) risk factors of GDM-1. Diabetes in the mother was an independent (*) risk factor of GDM-2.

The cumulative assessment of diabetes in the father and/or mother (and the grandfathers and/or grandmothers) was not related to the intensification of the risk of GDM. Horizontal lines represent (95%) confidence intervals (CI); the results for GDM-2 risk had wider confidence intervals, but the number of GDM-2 cases was small.

### 3.3. Risk of GDM (-1 and -2) after Cohort Dissection into Pre-pregnancy BMI Categories

Table 8 shows the adjusted odds ratios of gestational diabetes mellitus (GDM, GDM-1 and GDM-2) for declared family history of diabetes in the parents, calculated in the subgroups of pre-pregnancy BMI categories (obesity is the main modifiable risk factor of GDM). The results were adjusted (in multiple logistic regression) in model-a because the sizes of the subgroups were smaller compared to the whole cohort.

Diabetes in the father was an independent risk factor of GDM in the whole cohort and the subgroup of pregnant women with normal BMI. Importantly, overweight and/or obesity exacerbated the relationships studied, and the risk of GDM was approximately twice as high as compared to the results of pregnant women with normal BMI.

Interestingly, the results in Table 8 suggest a significant influence of diabetes in the father on the risk of GDM, even in lean pregnant women. In the underweight subgroup, the odds ratio of GDM for diabetes in the father was also higher, but this subgroup is very small and the confidence interval is wider, therefore, the result should be examined in a larger cohort.

Table S2 shows the risk of GDM (-1 and -2) for a family history of diabetes in the grandparents, calculated in the subgroups of pre-pregnancy BMI categories (in multiple logistic regression). Compared to ‘absence of diabetes in the family’, women with ‘diabetes in the grandmother’ had about a 2-fold increase in GDM-1 risk in the whole cohort and subgroup of normal BMI. The odds ratio of GDM-1 increased in the subgroup of women who were overweight (AOR-a = 4.79 (0.73–31.63); *p* = 0.104). This result is statistically insignificant, but this subgroup is very small, and the confidence interval is wider.

## 4. Discussion

The results of this study showed significant and independent associations (independent from the influence of other risk factors) between the categories of the family history of diabetes and the risk of each form of gestational diabetes mellitus (GDM), also pointing to differences. Diabetes in the father, mother or grandmother(s) were the independent risk factors of GDM-1, and diabetes in the mother of pregnant women was the independent risk factor of GDM-2 (also in the subgroup of pregnant women with normal BMI).

The cumulative assessment of diabetes in the father and mother (or grandmothers and grandfathers) was not related to the intensification of the risk of GDM. However, the presence of diabetes mellitus in both the mother and grandmothers was associated with a significant increase in the risk of GDM-1 diabetes.

Importantly, the results of this study also showed that pre-pregnancy obesity and/or overweight exacerbated the relationships studied. When categories of family history of diabetes coexisted with pre-pregnancy obesity/overweight, the risk of GDM was approximately twice as high as compared to the results of pregnant women with normal BMI. However, the results obtained in the subgroups had wider confidence intervals, therefore caution is advised in their interpretation.

The results of this study have important clinical implications; they indicate and emphasize the need for an intensive search for lifestyle interventions in order to normalize women’s weight (BMI) before pregnancy to reduce the risk of developing GDM.

This study brings added value to the existing literature. Unlike numerous retrospective case–control studies, this was a prospective cohort study. Our study separately assessed the risk of GDM-1 and GDM-2 for the defined categories of family history of diabetes. The results of this study were adjusted, and analyses were also performed by subgroups. Our cohort was Caucasian and included no chronic disease cases, such as pre-existing diabetes or hypertension.

In the available literature, many studies found a higher risk of GDM for family history of diabetes, as meta-analyses have shown [24,35], however, insignificant results were also found, and ‘family diabetes’ were assessed as a single variable. Rhee et al. found a statistically significantly higher risk of GDM for paternal diabetes and (separately) for maternal diabetes, and the results for paternal history was higher [34]. Tabak et al. found a statistically significantly higher risk of GDM for maternal diabetes and for the maternal line [32]. However, these studies did not evaluate separate GDM-1 and -2. Interestingly, Kong et al., examining insulinogenic indices and insulin sensitivity indexes in offspring, found statistically significant relationships with paternal and maternal history of diabetes, but they also found differences [38]. This suggests that paternal and maternal factors may participate in the development of diabetes in the offspring, but through different mechanisms.

The general characteristics of the participants of the current study are consistent with the results in the literature [11]. In the entire cohort, the percentage of women who developed GDM was 16%. Women with pre-pregnancy BMI ≥ 25 kg/m^2^ were 29.7%, and those with obesity were 10.8%. Women developing GDM (vs. non-diabetic women) were statistically significantly older, and the percentage of women with pre-pregnancy obesity was statistically significantly higher (21.9% vs. 8.6%). In the GDM group, a statistically significantly lower gestational age of newborns and a higher percentage of macrosomia were found (Table 2).

Thus far, no unambiguous biochemical markers for the prediction of gestational diabetes mellitus (GDM) have been identified, and extensive screening tests (including clinical and biochemical factors) are complex and have not been validated in external cohorts [4,11], but the etiology of GDM is complicated and is not fully understood [4]. Therefore, clinical risk factors (also identified in this study) may play an important role in the early prediction of GDM, allowing for the early implementation of surveillance and prevention [13]. It is important to affirm the role of the family history of diabetes as an independent risk factor for GDM-1 and -2, as this information is available in early pregnancy and even before pregnancy. Thus far, many candidate genes have been identified, although not all the results are conclusive and they require further investigation [26,27,28,29,30,31,39].

This study showed the role of obesity and overweight as factors that can modify (intensify) the influence of genetic factors. Since obesity was also an independent risk factor for GDM-1 and GDM-2 (Table 3), the goal of the campaigns (including those addressed to individuals and social groups) should be to optimize weight in women before pregnancy by promoting healthy lifestyles, i.e., by optimizing nutrition and physical activity.

National and international scientific societies issue guidelines for proper nutrition of pregnant women, as described in our previous work [40]. Research has shown that many dietary factors prior to pregnancy increase the risk of GDM, including sweets, animal fat and protein, high consumption of red and processed meat, as well as high consumption of fries and refined grains. In contrast, the beneficial effects of the Mediterranean diet, green vegetables, fruit, fish, poultry or fiber are well known [10,41,42]. Intervention studies to date have shown that early implementation of dietary and physical activity interventions (in the first trimester) may reduce the risk of GDM. At the same time, studies confirm that the diagnosis and treatment of GDM (by modulating diet, promoting physical activity and pharmacotherapy including insulin) may improve results in the mother and the child [10,11,43,44,45].

### Advantages and Limitations

The strength of this analysis was the prospective model of the study (categories of family history were reported prior to development of pregnancy complications). An advantage is researching the categories of family diabetes separately. A strength is the separate assessment of the GDM-1 and GDM-2 risks. In this study, results were adjusted for many confounders, although there may be other risk factors that would influence our results. Additionally, analyses after stratification into categories of pre-pregnancy BMI (and smoking) were conducted. Family history was reported by women in the questionnaires, and it was also cross-checked with the data in the medical reports.

The results obtained in the subgroups and for GDM-2 risk had wider confidence intervals, which requires caution in the interpretation. However, these results were statistically significant, indicating that they were not accidental. These wider confidence intervals were likely due to small subgroup sizes, and therefore, the results of these reports should be checked in a larger sample size.

The family history of diabetes was self-reported, and details of the history were not known (e.g., no information on the type of diabetes, T1D or T2D, in the parents or grandparents), however, this is a typical limitation of analyses on this topic.

In this analysis, detailed information on GDM (e.g., the degree of glycemic control during pregnancy) was not known for each case (data were extracted from medical records of women admitted to childbirth).

This study was a single-center study and may not reflect national statistics. The study was conducted in a high-level referral center attended by women with risk factors, which could increase the proportion of GDM cases in the cohort.

Pre-pregnancy weight was self-reported, which is a typical limitation.

The information from the questionnaires (pre-pregnancy weight, smoking, the family history and more) were also verified with the data from the medical records and with the data from an additional questionnaire (collected after the 12th week of puerperium).

## 5. Conclusions

The results of this study showed that certain categories of the family history of diabetes were independent risk factors for gestational diabetes mellitus (GDM) (also in the subgroup of pregnant women with normal BMI). Apart from the maternal influence of diabetes, the results suggest a significant influence of diabetes in the father on the risk of GDM, even (interestingly) in lean pregnant women. This is an area for future study.

Importantly, pre-pregnancy obesity and/or overweight intensified the relationships studied, suggesting that the ongoing goal should be to seek interventions (in the area of education and lifestyle, diet and exercise) to optimize women’s weight before pregnancy. These activities can promote the health of the mother and the child. The results of this study suggest that shared (family) lifestyle or susceptibility to obesity is likely to contribute to the studied effects.

In this study, the subgroups were smaller, and the results obtained had broad confidence intervals, therefore, the results of these reports should be examined in a larger cohort. A further interesting analysis would be if the family history influences the glucose concentrations at a certain timepoint in the OGTT (i.e., does a family history predispose to increasing post-load hyperglycemia, or does it have an equal influence on all timepoints?).

## Figures and Tables

**Table 1:** Characteristics of the covariates.

Covariates	Definitions and Categories
Maternal age	Maternal age was taken from medical reports. This variable was defined as completed maternal age at conception; in years. Maternal age as a continuous variable was a covariate.
Pre-pregnancy BMI (body mass index)	Pre-pregnancy BMI was self-reported in the Questionnaire This variable was defined as the quotient of pre-pregnancy weight (kg) and height (meters) squared. BMI as a continuous variable was a covariate. BMI was assessed in the five following categories as subgroups: (1) normal BMI (18.5–24.9); (2) underweight (<18.5); (3) overweight (25.0–29.9); (4) obesity (≥30); and (5) BMI ≥ 25 kg/m^2^.
GWG (gestational weight gain)	GWG was based on maternal weight before childbirth (from medical reports) and pre-pregnancy weight (self-reported). This variable was defined as the difference between the maternal weight before childbirth and the weight before pregnancy. GWG was assessed in the three following categories regardless of pre-pregnancy BMI categories (according to the Institute of Medicine (IOM) recommendations from 2009): (1) GWG in the range; (2) GWG above the range; and (3) GWG below the range. GWG out of the range was a covariate.
Multiparity	Parity was taken from medical records. The two following categories of parity were taken into consideration: (1) multiparity (≥1 prior childbirths) and (2) primiparity (zero prior childbirth). Multiparity was a covariate.
Smoking	Smoking status was self-reported in the Questionnaire. Smoking was assessed in the three following categories as subgroups: (1) women who had never smoked; (2) women who smoked before pregnancy); and (3) women who smoked in the first trimester. The category of ‘smokers in the first trimester’ was a covariate.
Prior GDM	The maternal history was taken from medical reports. This variable was defined as gestational diabetes mellitus (GDM) in previous pregnancies. The two following categories were taken into consideration: (1) prior GDM; (2) no prior GDM. Prior GDM was a covariate.

GDM: gestational diabetes mellitus.

**Table 2:** Basic characteristics of the participants developing gestational diabetes mellitus (GDM).

Maternal Characteristics	Non-Diabetic Group (*n* = 766)	GDM Group (*n* = 146)	*p* *
	Median (IQR), *n* (%)	Median (IQR), *n* (%)	
Basic characteristics			
Maternal age (years)	34.0 (30.0–37.0)	36.0 (33.0–38.0)	<0.001
Pre-pregnancy BMI (kg/m^2^)	22.6 (20.4–25.7)	24.0 (21.5–29.1)	<0.001
Categories:			<0.001
Obesity	66 (8.6%)	32 (21.9%)	
Overweight	147 (19.2%)	26 (17.8%)	
Normal BMI	515 (67.2%)	79 (54.1%)	
Underweight	38 (5.0%)	9 (6.2%)	
GWG (kg)	14.0 (11.0–17.0)	10.0 (7.0–15.0)	<0.001
Primiparity	318 (41.5%)	64 (43.8%)	0.602
Smokers in the 1st tr.	47 (6.1%)	10 (6.8%)	0.744
Prior GDM	3 (0.4%)	8 (5.5%)	<0.001
Pregnancy outcomes			
Fetal sex—son	391 (51.0%)	82 (56.2%)	0.256
Gestational age (weeks)	39.0 (38.0–40.0)	39.0 (38.0–39.8)	0.032
Birth weight (g)	3400.0 (3082.5–3707.5)	3400.0 (3065.0–3780.0)	0.577
Categories:			0.047
Birth weight < 2500 g	54 (7.0%)	6 (4.1%)	
Birth weight 2500–4000 g	636 (83.0%)	119 (81.5%)	
Birth weight > 4000 g	76 (9.9%)	21 (14.4%)	
PIH	112 (14.6%)	25 (17.1%)	0.438

* The Mann–Whitney U test was applied for comparisons of continuous variables (the variables were not normally distributed) and the Pearson chi-square test was applied (or Fisher exact test when Cochran assumption was not met) for comparisons of binomial variables, and the Cochran–Armitage test was applied for comparisons of trend for categorical variables (*p* < 0.05 was assumed to be significant). GDM: gestational diabetes mellitus (125 cases of GDM-1 and 21 cases of GDM-2); BMI: body mass index; GWG: gestational weight gain; PIH: pregnancy-induced hypertension.

**Table 3:** The odds ratios of gestational diabetes mellitus (GDM -1, -2) for the basic risk factors.

Basic Risk Factors	GDM-1 Risk	GDM-2 Risk
	AOR-A (95% CI); *p* *	AOR-A (95% CI); *p* *
Pre-pregnancy BMI (kg/m^2^):		
Obesity (≥30)	2.27 (1.32–3.91); 0.003	6.91 (2.38–20.05); <0.001
Overweight (25–29.9)	0.95 (0.56–1.61); 0.845	2.01 (0.64–6.38); 0.234
Underweight (<18.5)	1.74 (0.76–3.96); 0.187	2.33 (0.28–19.67); 0.436
Normal BMI (18.5–24.9)		1
Smoking in the 1st tr.	1.34 (0.64–2.81); 0.434	-
Smoking before pregnancy	0.63 (0.32–1.23); 0.175	0.61 (0.14–2.76); 0.525
Never smoked	1	1
GWG above the range	0.68 (0.41–1.13); 0.137	0.51 (0.16–1.67); 0.267
GWG below the range	2.39 (1.5–3.82); <0.001	2.99 (1.03–8.74); 0.045
GWG in the range	1	1
Maternal age (years):		
≥40	2.31 (0.99–5.34); 0.052	1.20 (0.22–6.75); 0.832
18–24	0.43 (0.09–2.00); 0.283	-
25–29	1	1
Prior GDM	9.88 (1.87–52.29); 0.007	138.7 (23.7–812.8); <0.001
No prior GDM	1	1
Multiparity	0.57 (0.38–0.86); 0.007	1.91 (0.66–5.53); 0.230
Primiparity	1	1
Preeclampsia	0.21 (0.03–1.64); 0.138	2.21 (0.40–12.08); 0.360
No PIH	1	1

* AOR-a: adjusted odds ratios (95% confidence intervals) calculated in multiple logistic regression (model-a) after adjusted for multiparity, pre-pregnancy BMI, maternal age, gestational weight gain (GWG) out of the range as well as smoking in the first trimester (*p*-value < 0.05 was assumed to be significant) (the examined risk factor was excluded from the confounding variables mentioned). GDM-1: gestational diabetes mellitus treated with diet (*n* = 125); GDM-2: gestational diabetes mellitus treated with insulin (*n* = 21); Controls: non-diabetic women (*n* = 766).

**Table 4:** The characteristics of declared family history of diabetes.

Maternal Characteristics	Non-Diabetic Group (*n* = 766)	GDM Group (*n* = 146)	*p* *
	*n* (%)	*n* (%)	
Diabetes in Family **			
-in the mother	52 (6.8%)	18 (12.5%)	0.019
-in the father	74 (9.7%)	36 (25.0%)	<0.001
-in both parents simultaneously	14 (1.8%)	4 (2.8%)	0.510
-in the grandmother(s)	70 (9.2%)	22 (15.3%)	0.030
-in the grandfather(s)	37 (4.8%)	6 (4.2%)	0.724
-in both grandparents simultaneously	8 (1.0%)	1 (0.7%)	1
-in both mother and grandmother(s) simultaneously	2 (0.3%)	2 (1.4%)	0.122
-in both father and grandfather(s) simultaneously	6 (0.8%)	1 (0.7%)	1

* The Pearson chi-square test was applied (or Fisher exact test when Cochran assumption was not met) for comparisons of binomial variables, and the Cochran–Armitage test was applied for comparisons of trend for categorical variables (*p* < 0.05 was assumed to be significant); ** Analyses for available data. GDM: gestational diabetes mellitus (125 cases of GDM-1 and 21 cases of GDM-2).

**Table 5:** The adjusted odds ratios of gestational diabetes mellitus (GDM-1, -2) for declared diabetes in the parents.

Risk Factors/ Diabetes in the Parents	Cases/ Controls	OR (95% CI); *p*	AOR-A (95% CI); *p* *	AOR-B (95% CI); *p* *
GDM-1 risk				
-in the father	32/74	3.90 (2.39–6.37); <0.001	3.71 (2.25–6.12); <0.001	3.68 (2.23–6.07); <0.001
-in the mother	14/52	2.43 (1.27–4.63); 0.007	2.13 (1.1–4.14); 0.026	2.13 (1.1–4.14); 0.026
-in the mother or father	42/112	3.38 (2.18–5.26); <0.001	3.21 (2.05–5.04); <0.001	3.20 (2.03–5.02); <0.001
-in both parents simultaneously	4/14	2.58 (0.82–8.07); 0.104	1.88 (0.58–6.14); 0.293	1.88 (0.58–6.13); 0.294
Ref **	62/559	1	1	1
GDM-2 risk				
-in the father	4/74	2.75 (0.85–8.85); 0.09	2.93 (0.87–9.88); 0.083	3.16 (0.78–12.81); 0.108
-in the mother	4/52	3.91 (1.2–12.71); 0.023	3.36 (0.97–11.62); 0.056	4.73 (1.26–17.77); 0.021
-in the mother or father	8/112	3.63 (1.43–9.23); 0.007	3.76 (1.42–9.99); 0.008	4.77 (1.59–14.25); 0.005
-in both parents simultaneously	0/14	-	-	-
Ref **	11/559	1	1	1
GDM (all cases) risk				
-in the father	36/74	3.73 (2.34–5.94); <0.001	3.51 (2.18–5.66); <0.001	3.49 (2.15–5.67); <0.001
-in the mother	18/52	2.65 (1.47–4.78); 0.001	2.24 (1.22–4.12); 0.009	2.32 (1.26–4.28); 0.007
-in the mother or father	50/112	3.42 (2.26–5.17); <0.001	3.2 (2.09–4.89); <0.001	3.23 (2.1–4.97); <0.001
-in both parents simultaneously	4/14	2.19 (0.7–6.82); 0.177	1.49 (0.46–4.82); 0.509	1.51 (0.46–4.91); 0.494
Ref **	73/559	1	1	1

* AOR: adjusted odds ratios (95% confidence intervals) calculated in multiple logistic regression after adjusted for multiparity, pre-pregnancy BMI, age, gestational weight gain (GWG) out of the range as well as smoking in the first trimester (AOR-a), plus prior gestational diabetes mellitus (AOR-b) (*p*-value < 0.05 was assumed to be significant). ** Reference category: ‘Absence of diabetes in the family’. Cases: GDM-1, i.e., diabetes treated with diet (*n* = 125); GDM-2, i.e., diabetes treated with insulin (*n* = 21). Controls: non-diabetic women (*n* = 766).

**Table 6:** The adjusted odds ratios of gestational diabetes mellitus (GDM-1,-2) for declared diabetes in the grandparents.

Risk Factors/ Diabetes in the Grandparents	Cases/ Controls	OR (95% CI); *p*	AOR-A (95% CI); *p* *	AOR-B (95% CI); *p* *
GDM-1 risk				
-in the grandfather(s)	5/37	1.22 (0.46–3.21); 0.690	1.26 (0.47–3.36); 0.647	1.28 (0.48–3.41); 0.626
-in the grandmother(s)	19/70	2.45 (1.38–4.33); 0.002	2.48 (1.38–4.47); 0.002	2.34 (1.29–4.24); 0.005
-in the grandfathers or grandmothers	23/99	2.09 (1.24–3.54); 0.006	2.18 (1.28–3.73); 0.004	2.07 (1.21–3.57); 0.008
-in both simultaneously	1/8	1.13 (0.14–9.16); 0.911	1.15 (0.14–9.59); 0.896	1.15 (0.14–9.56); 0.898
Ref **	62/559	1	1	1
GDM-2 risk				
-in the grandfather(s)	1/37	1.37 (0.17–10.93); 0.764	1.59 (0.18–13.62); 0.674	1.21 (0.12–12.29); 0.870
-in the grandmother(s)	3/70	2.18 (0.59–8.00); 0.241	1.71 (0.44–6.67); 0.440	1.55 (0.33–7.42); 0.581
-in the grandfathers or grandmothers	4/99	2.05 (0.64–6.58); 0.226	1.82 (0.54–6.07); 0.332	1.53 (0.4–5.87); 0.539
-in both simultaneously	0/8	-	-	-
Ref **	11/559	1	1	1

* AOR: adjusted odds ratios (95% confidence intervals) calculated in multiple logistic regression after adjusted for multiparity, pre-pregnancy BMI, age, gestational weight gain (GWG) out of the range as well as smoking in the first trimester (AOR-a), plus prior gestational diabetes mellitus (AOR-b) (*p*-value < 0.05 was assumed to be significant). ** Reference category: ‘Absence of diabetes in the family’. Cases: GDM-1, i.e., diabetes treated with diet (*n* = 125); GDM-2, i.e., diabetes treated with insulin (*n* = 21). Controls: non-diabetic women (*n* = 766).

**Table 7:** The adjusted odds ratios of gestational diabetes mellitus (GDM-1,-2) for declared diabetes in the parents and grandparents.

Risk Factors/ Diabetes in Family	Cases/ Controls	OR (95% CI); *p*	AOR-A (95% CI); *p* *	AOR-B (95% CI); *p* *
GDM-1 risk				
-in both mother and grandmothers simultaneously	2/2	9.02 (1.25–65.13); 0.029	8.85 (1.17–66.9); 0.035	8.80 (1.16–66.57); 0.035
-in the mother or grandmothers	31/120	2.33 (1.45–3.74); <0.001	2.22 (1.36–3.61); 0.001	2.14 (1.31–3.50); 0.002
Ref **	62/559	1	1	1
-in both father and grandfathers simultaneously	1/6	1.5 (0.18–12.69); 0.708	1.41 (0.16–12.14); 0.753	1.41 (0.16–12.12); 0.754
-in the father or grandfathers	36/105	3.09 (1.95–4.9); <0.001	3.08 (1.92–4.92); <0.001	3.05 (1.9–4.88); <0.001
Ref **	62/559	1	1	1
GDM-2 risk				
-in both mother and grandmothers simultaneously	0/2	-	-	-
-in the mother or grandmothers	7/120	2.96 (1.13–7.8); 0.028	2.46 (0.9–6.72); 0.080	2.88 (0.95–8.76); 0.062
Ref **	11/559	1	1	1
-in both father and grandfathers simultaneously	0/6	-	-	-
-in the father or grandfathers	5/105	2.42 (0.82–7.11); 0.108	2.54 (0.84–7.7); 0.099	2.29 (0.67–7.89); 0.188
Ref **	11/559	1	1	1

* AOR: adjusted odds ratios (95% confidence intervals) calculated in multiple logistic regression after adjusted for multiparity, pre-pregnancy BMI, age, gestational weight gain (GWG) out of the range as well as smoking in the first trimester (AOR-a), plus prior gestational diabetes mellitus (AOR-b) (*p*-value < 0.05 was assumed to be significant). ** Reference category: ‘Absence of diabetes in the family’. Cases: GDM-1, i.e., diabetes treated with diet (*n* = 125); GDM-2, i.e., diabetes treated with insulin (*n* = 21). Controls: non-diabetic women (*n* = 766).

**Table 8:** The adjusted odds ratios of gestational diabetes mellitus (GDM-1, -2) for declared diabetes in the parents, in the subgroups of BMI categories.

Risk Factors/ Diabetes in the Parents	GDM Risk	GDM-1 Risk	GDM-2 Risk
	AOR-A (95% CI); *p* *	AOR-A (95% CI); *p* *	AOR-A (95% CI); *p* *
Whole cohort			
-in the father	3.51 (2.18–5.66); <0.001	3.71 (2.25–6.12); <0.001	2.93 (0.87–9.88); 0.083
-in the mother	2.24 (1.22–4.12); 0.009	2.13 (1.1–4.14); 0.026	3.36 (0.97–11.62); 0.056
Ref **	1	1	1
Normal BMI			
-in the father	3.34 (1.75–6.39); <0.001	3.24 (1.65–6.38); 0.001	3.82 (0.67–21.77); 0.131
-in the mother	1.67 (0.67–4.19); 0.270	1.63 (0.62–4.29); 0.324	1.64 (0.15–17.8); 0.684
Ref **	1	1	1
Underweight			
-in the father	2.23 (0.26–18.95); 0.462	2.46 (0.29–20.89); 0.41	-
-in the mother	-	-	-
Ref **	1	1	1
Overweight			
-in the father	6.24 (1.87–20.79); 0.003	6.23 (1.7–22.84); 0.006	5.11 (0.39–66.95); 0.214
-in the mother	3.7 (0.86–15.93); 0.079	2.94 (0.55–15.6); 0.206	6.91 (0.48–100.53); 0.157
Ref **	1	1	1
Obesity			
-in the father	4.57 (1.29–16.2); 0.019	6.66 (1.57–28.36); 0.01	2.16 (0.17–27.47); 0.554
-in the mother	3.6 (0.99–12.99); 0.051	3.86 (0.89–16.72); 0.071	6.49 (0.6–70.53); 0.124
Ref **	1	1	1
BMI ≥ 25 kg/m^2^			
-in the father	4.36 (1.97–9.64); <0.001	4.89 (2.08–11.48); <0.001	2.73 (0.48–15.37); 0.256
-in the mother	3.84 (1.58–9.35); 0.003	3.38 (1.25–9.2); 0.017	5.56 (1.18–26.24); 0.03
Ref **	1	1	1

* AOR-a: adjusted odds ratios (with 95% confidence intervals, CI) calculated in multiple logistic regression (model-a) after adjusted for multiparity, pre-pregnancy BMI, maternal age, gestational weight gain (GWG) out of the range as well as smoking in the first trimester (*p*-value < 0.05 was assumed to be significant). ** Reference category: ‘Absence of diabetes in the family’. GDM-1: gestational diabetes mellitus treated with diet (*n* = 125); GDM-2: gestational diabetes mellitus treated with insulin (*n* = 21); Controls: non-diabetic women (*n* = 766).

## Data Availability

The data presented in this study are available on request from the corresponding author. The data are not publicly available as it contains a variety of patient information and covers a much wider range than needed for the analyzes presented here.

## Supplementary Materials

The following are available online at https://www.mdpi.com/article/10.3390/ijerph18136936/s1, Table S1: Odds ratios of gestational diabetes mellitus (GDM-1, -2) for basic risk factors (set of data), Table S2: Odds ratios of gestational diabetes mellitus (GDM-1, -2) for declared diabetes in grandparents, in the subgroups of pre-pregnancy BMI categories.

## Abbreviations

AOR	Adjusted odds ratio
BMI	Body mass index
CI	Confidence intervals
DOHaD	Developmental Origins of Health and Disease
GDM	Gestational diabetes mellitus
GWG	Gestational weight gain
IOM	Institute of Medicine
OGTT	Oral glucose tolerance test
OR	Odds ratio
T2D	Type 2 diabetes
PE	Preeclampsia
PIH	Pregnancy-induced hypertension
WHO	World Health Organization
