# Supplementary material for: Gestational Diabetes Mellitus (GDM) Risk for Declared Family History of Diabetes, in Combination with BMI Categories
Source: Int J Environ Res Public Health. 2021 Jun 28;18(13):6936. doi: 10.3390/ijerph18136936 (PMC8293805; doi:10.3390/ijerph18136936)
Supplement: Supplementary file 1 [file ijerph-18-06936-s001.zip › Table S1.pdf]

Table S1. Odds ratios of gestational diabetes mellitus (GDM -1, -2) for basic risk factors (set of data).

Risk factors	Cases/ Controls	OR (95% CI), p	AOR-a (95% CI), p
		**GDM-1 risk**	
Pre-pregnancy BMI, kg / m²):			
Obesity (≥ 30)	25/66	2.75 (1.63−4.63); <0.001	2.27 (1.32−3.91); 0.003
Overweight (24.5−29.9)	21/147	1.04 (0.62−1.74); 0.893	0.95 (0.56−1.61); 0.845
Underweight (<18.5)	8/38	1.53 (0.69−3.41); 0.301	1.74 (0.76-3.96); 0.187
Normal BMI (18.5−24.9)	71/515	1	1
Smoking in the 1st tr.	10/47	1.33 (0.65−2.71); 0.431	1.34 (0.64−2.81); 0.434
Smoking before pregn.	21/145	0.86 (0.52−1.43); 0.571	0.63 (0.32−1.23); 0.175
Never smoked	104/621	1	1
GWG above the range	33/298	0.83 (0.51−1.35); 0.454	0.68 (0.41−1.13); 0.137
GWG in the range	39/292	1	1
GWG below the range	53/176	2.25 (1.43−3.55); <0.001	2.39 (1.5−3.82); <0.001
Maternal age, years:			
≥ 40	14/59	2.15 (0.96−4.8); 0.061	2.31 (0.99−5.34); 0.052
25−29	14/127	1	1
18−24	2/38	0.48 (0.1−2.19); 0.342	0.43 (0.09−2); 0.283
Prior GDM	3/3	6.25 (1.25−31.34); 0.026	9.88 (1.87−52.29); 0.007
No prior GDM	122/763	1	1
Multiparity	66/448	0.79 (0.54−1.16); 0.234	0.57 (0.38−0.86); 0.007
Primiparity	59/318	1	1
PE	1/21	0.3 (0.04−2.23); 0.238	0.21 (0.03−1.64); 0.138
No PIH	105/654	1	1
		**GDM−2 risk**	
Pre-pregnancy BMI (kg / m²):			
Obesity (≥ 30)	7/66	6.83 (2.4−19.44); <0.001	6.91 (2.38−20.05); <0.001
Overweight (24.5−29.9)	5/147	2.19 (0.17−6.79); 0.175	2.01 (0.64−6.38); 0.234
Underweight (<18.8)	1/38	1.69 (0.21−13.9); 0.624	2.33 (0.28-19.67); 0.436
Normal BMI (18.5−24.9)	8/515	1	1
Smoking in the 1st tr.	0/47	−	−
Smoking before pregn.	2/145	0.45 (0.1−1.96); 0.288	0.61 (0.14−2.76); 0.525
Never smoked	19/621	1	1
GWG above the range	5/298	0.7 (0.22−2.23); 0.546	0.51 (0.16−1.67); 0.267
GWG in the range	7/292	1	1
GWG below the range	9/176	2.13 (0.78−5.83); 0.140	2.99 (1.03−8.74); 0.045
Maternal age, years:			
≥ 40	3/59	2.15 (0.42−10.98); 0.357	1.2 (0.22−6.75); 0.832
25−29	3/127	1	1
18−24	0/38	−	−
Prior GDM	5/3	79.48 (17.48−361.43); <0.001	138.71 (23.67−812.84); <0.001
No prior GDM	16/763	1	1
Multiparity	16/448	2.27 (0.82−6.26); 0.113	1.91 (0.66−5.53); 0.230
Primiparity	5/318	1	1
PE	2/21	3.89 (0.84−18.03); 0.082	2.21 (0.4−12.08); 0.360
No PIH	16/654	1	1

* AOR-a: adjusted odds ratios (with 95% confidence intervals, CI) calculated in multiple logistic regression (model-a) after adjusted for primiparity, maternal age, pre-pregnancy BMI, gestational weight gain (GWG) out of the range as well as smoking in the first trimester (p- value < 0.05 was assumed to be significant) (the examined risk factor was excluded from the confounding variables mentioned). Cases: GDM-1i.e. gestational diabetes mellitus treated with diet (*n* = 125); GDM-2 i.e. gestational diabetes mellitus treated with insulin (*n* = 21); Controls: non-diabetic women (*n* = 766).
