# Supplementary material for: Gestational Diabetes Mellitus (GDM) Risk for Declared Family History of Diabetes, in Combination with BMI Categories
Source: Int J Environ Res Public Health. 2021 Jun 28;18(13):6936. doi: 10.3390/ijerph18136936 (PMC8293805; doi:10.3390/ijerph18136936)
Supplement: Supplementary file 1 [file ijerph-18-06936-s001.zip › Table S2.pdf]

Table S2. Odds ratios of gestational diabetes mellitus (GDM -1, -2) for declared diabetes in grandparents, in the subgroups of pre-pregnancy BMI categories.

Risk factors / Diabetes in GRANDPARENTS	GDM-1 risk		GDM-2 risk	
	**Cases / Controls**	**AOR-a (95% CI); *p* ***	**Cases / Controls**	**AOR-a (95% CI); *p* ***
Whole cohort				
- in the grandfather	5/37	1.26 (0.47-3.36); 0.647	1/37	1.59 (0.18-13.62); 0.674
- in the grandmother	19/70	2.48 (1.38-4.47); 0.002	3/70	1.71 (0.44-6.67); 0.440
Ref. **	62/559	1	11/559	1
Normal BMI				
- in the grandfather	4/25	1.7 (0.55-5.24); 0.358	1/25	3.41 (0.33-35.14); 0.302
- in the grandmother	11/44	2.68 (1.25-5.75); 0.011	2/44	4.83 (0.83-28.17); 0.080
Ref. **	39/383	1	4/383	1
Underweight				
- in the grandfather	0/2	-	0/2	-
- in the grandmother	1/5	0.9 (0.06-14.44); 0.940	0/5	-
Ref. **	5/24	1	1/24	1
Overweight				
- in the grandfather	1/7	4.21 (0.36-49.06); 0.252	0/7	-
- in the grandmother	3/9	4.79 (0.73-31.63); 0.104	1/9	7.69 (0.37-157.58); 0.186
Ref. **	9/112	1	2/112	1
Obesity				
- in the grandfather	0/3	-	0/3	-
- in the grandmother	4/12	1.46 (0.31-6.95); 0.633	0/12	-
Ref. **	9/40	1	4/40	1
BMI ≥ 25 kg / m^2				
- in the grandfather	1/10	0.82 (0.09-7.13); 0.855	0/10	-
- in the grandmother	7/21	2.96 (1.05-8.32); 0.040	1/21	1.09 (0.12-9.9); 0.940
Ref. **	18/152	1	6/152	1

* AOR-a: adjusted odds ratios (with 95% confidence intervals, CI) calculated in multiple logistic regression (model-a) after adjusted for multiparity, maternal age, pre-pregnancy BMI, gestational weight gain (GWG) out of the range as well as smoking in the first trimester (p- value < 0.05 was assumed to be significant). ** Reference category: 'Absence of diabetes in the family'. Cases: GDM-1 i.e. gestational diabetes mellitus treated with diet (n = 125); GDM-2 i.e. gestational diabetes mellitus treated with insulin (n = 21); Controls: non-diabetic women (n = 766).
